# A community-based assessment of correlates of facility delivery among HIV-infected women in western Kenya

**DOI:** 10.1186/s12884-015-0467-6

**Published:** 2015-02-25

**Authors:** John Kinuthia, Pamela Kohler, John Okanda, George Otieno, Frank Odhiambo, Grace John-Stewart

**Affiliations:** Department of Research and Programs/Department of Reproductive Health Kenyatta National Hospital, Nairobi, Kenya; Global Health and Psychosocial and Community Health, University of Washington, Seattle, WA USA; Center for Global Health Research, Kenya Medical Research Institute, Kisumu, Kenya; Global Health, Medicine, Pediatrics, and Epidemiology, University of Washington, Seattle, WA USA

**Keywords:** HIV, Facility delivery, Infant, Partner, Antiretroviral

## Abstract

**Background:**

Childbirth at health facilities is an important strategy to reduce maternal morbidity and mortality, improve fetal outcomes, and reduce mother-to-child transmission of HIV. Although access to antenatal care in Kenya is high (>90%), less than half of births occur at health facilities. This analysis aims to assess correlates of facility delivery among recently pregnant HIV-infected women participating in a community-based survey, and to determine whether these correlates were unique when compared to HIV-uninfected women from the same region.

**Methods:**

Women residing in the Kenya Medical Research Institute/Centers for Disease Control and Prevention Health and Demographic Surveillance System, and who had delivered an infant in the previous year were visited at home in 2011. Consenting mothers answered a questionnaire assessing demographics, place of delivery, utilization of prevention of mother-to-child HIV transmission (PMTCT) services, and stigma indicators. Known HIV-positive women were purposively oversampled. Chi-square tests of proportions and multivariate logistic regression, stratified by HIV status, were performed to assess correlates of facility delivery.

**Results:**

Overall, 101 (46.8%) HIV-infected and 127 (39.9%) HIV-uninfected women delivered at health facilities. Among HIV-infected women, cost (42.8%), distance (18.8%) and fear of harsh treatment (15.2%) were primary disincentives for facility delivery; 2.9% noted fear of HIV testing was a disincentive. HIV-infected women who delivered at facilities had higher education (p = 0.04) and socioeconomic status (p < 0.005), initiated antenatal care (ANC) earlier (4.9 vs. 5.4 months, p = 0.016), were more likely to know partner’s HIV status (p = 0.016), report satisfaction with delivery care (p = 0.001) and use antiretrovirals (87.1% vs. 77.4%, p = 0.063) compared to those with non-facility delivery. Stigma indicators were not associated with delivery location. Similar cofactors of facility delivery were noted among uninfected women.

**Conclusions:**

Utilization of facility delivery remains low in Kenya and poses a challenge to elimination of infant HIV and reduction of peripartum mortality. Cost, distance, and harsh treatment were cited as barriers and these need to be addressed programmatically. HIV-infected women with lower socioeconomic status and those who present late to ANC should be prioritized for interventions to increase facility delivery. Partner involvement may increase use of maternity services and could be enhanced by couples counseling.

## Background

The Joint United Nations Programme on HIV/AIDS (UNAIDS) reports that there were ~260,000 children newly infected with HIV in 2012, most in sub-Saharan Africa [1]. Without interventions, ~30–45% of HIV-exposed infants become infected [2]. Optimal use of antiretroviral drugs (ARVs) reduces the risk of HIV transmission to <5% [3]. In 2011, the UNAIDS Global Plan for Virtual Elimination of Pediatric HIV was launched [4]. The initiative aimed to reduce the number of new pediatric HIV infections by 90% and the rate of mother-to-child transmission (MTCT) of HIV to 5% by 2015. Attaining this target requires that at least 90% of HIV-infected mothers utilize perinatal ARVs [4]. However, women who do not deliver at health facilities are less likely to use ARVs during delivery, a critical period when a substantial proportion of perinatally acquired HIV infections occur [5-7]. Additionally, women who do not deliver at health facilities fail to receive obstetric interventions to reduce risk of prolonged rupture of membranes and prolonged labor associated with increased rates of HIV transmission [8,9].

Every year, ~300,000 maternal deaths occur worldwide from conditions that are largely preventable or treatable [10-12]. Nearly all these deaths occur in developing countries. In 2008, World Health Organization estimated the life-time risk of maternal death for a woman in sub-Saharan Africa to be 1:31 compared to 1:4300 for a woman living in developed countries [13]. The huge disparity in the risk of maternal death is attributable to differences in utilization and quality of maternal health services. Although most obstetric complications occur around the time of delivery and cannot be predicted, they can be treated or prevented with appropriate obstetric interventions. Skilled attendance at delivery is thus advocated as the “single most important factor in preventing maternal deaths” [14]. However, skilled care at birth remains out of reach for many women in sub-Saharan Africa. The 5^th^ Millennium Development Goal (MDG5) aims to reduce maternal mortality ratio by 75% between 1990 and 2015 and targets a 90% facility delivery rate to achieve this goal [15].

The 2008 Kenya Demographic Health Survey (KDHS) reported that only 43% of mothers delivered in a health facility, a rate similar to 15 years earlier, demonstrating stagnant progress for this indicator [16]. The national maternal mortality ratio was estimated at 488 maternal deaths per 100,000 live births. HIV is a major cause of death among women of reproductive age, particularly in Africa, the region in which most (>90%) HIV-infected pregnant women reside [17]. In 2011, there were an estimated 273, 500 maternal deaths worldwide, approximately 20% of which were attributable to HIV [12]. However, a recent analysis estimated that HIV accounted for only 1.5% of maternal deaths in sub- Saharan in 2013. The authors postulated that reduced mortality attributable to HIV could be due to underestimates in HIV prevalence during pregnancy, underestimates in relative risk of death for a HIV-infected pregnant woman and impact of scale-up of antiretroviral therapy [11].

Ensuring that HIV-infected women deliver in health facility is therefore crucial to optimize maternal outcomes and to reduce risk of infant HIV infection. The aim of this nested study was to determine correlates of facility delivery among HIV-infected mothers in a rural community in Nyanza Province, western Kenya. Nyanza has the highest HIV prevalence (14.9%) in the country and is one of the regions with low rates (44%) of facility delivery [16,18]. Use of a population-based approach for this study provided the opportunity to access all recently delivered mothers, including those who did not utilize MCH services.

## Methods

### Study setting and population

This analysis utilized data obtained from a cross-sectional study of women residing in the Kenya Medical Research Institute/Centers for Disease Control and Prevention (KEMRI/CDC) Health and Demographic Surveillance System (HDSS) area who delivered an infant within a year prior to the survey. The KEMRI/CDC HDSS area is located northeast of Lake Victoria in the Nyanza Province of western Kenya [19]. The HDSS covers 385 villages with a population of approximately 220,000. The HDSS had implemented a program for home-based counseling and testing (HBCT) for HIV with high uptake of testing and linkage of HIV test result to HDSS dataset for all consenting participants. HIV-infected women were purposively oversampled to increase power of the study to detect associations related to uptake of PMTCT interventions.

### Recruitment and data collection

The methodology of the parent study has previously been described [20]. A list of women in the KEMRI-CDC HDSS area who had delivered in 2010 was generated. The sampling framework was designed to efficiently assess two populations relevant to PMTCT service delivery: a random sample of women in the general community to assess uptake of interventions targeting all pregnant women (ANC, HIV-testing), and a sample of known HIV positive women to assess uptake of interventions (antiretrovirals) targeting HIV-infected pregnant women. This included a comprehensive list of 275 women who had received their HIV status for at least 3 months before delivery and a second list of 523 randomly selected women from the areas where HBCT had not been conducted. Data generated from the HDSS included name, Global Positioning System location, and a randomly assigned identification code. Despite lead investigator knowledge of HIV status from the HDSS in HBCT areas for sampling purposes, field workers were blinded to HIV status and thus self-report of HIV status was used at interview.

Outcomes of interest included self-report of place of delivery. Cofactors assessed for place of delivery included age, education level, marital status, utilization of antenatal care (ANC), use of maternal and infant ARVs, satisfaction with care provided during delivery and knowledge of partner HIV status. Socioeconomic status was assessed by using indicators such ownership of mobile phones, radio, bicycle, cattle and monthly family income. Standardized questions were used to quantitatively measure HIV-related stigma and discrimination [21]. We evaluated two domains of HIV stigma, namely: value- and morality-related attitudes of blame, judgment and shame for those living with HIV/AIDS; and enacted stigma or discrimination. To assess perceptions of community behavior, or perhaps reasons that women were unwilling to report themselves about reasons for non-facility delivery, all participants were also asked an open-ended question, “In your opinion, what are some of the reasons women in this area do not deliver their babies in a health facility?”.

### Data analysis

Analyses were restricted to women self-reporting HIV-positive status or HIV-negative status. Those who reported not knowing their status, and those who reported they were negative but who were known to be HIV-positive through prior HBCT, were excluded. STATA version 10 (STATA Corp, College Station, Texas, USA) was used to analyze data on rates and correlates of facility delivery and association of facility delivery with use of maternal and infant antiretrovirals. We used Pearson’s Chi square or Fisher’s exact tests to compare categorical variables, and t-tests were used for continuous variables. Multivariate logistic regression was conducted using covariates statistically associated (p < 0.05) with facility delivery in univariate analysis.

### Ethical approval

Approval for the study was obtained from the Kenya Medical Research Institute (KEMRI) Ethical Review Committee (ERC), and from the Human Subjects Division at the University of Washington. Authorization was also obtained from Provincial Medical Officer, Nyanza and the District Medical Officers of Health. Study participants provided written informed consent to be interviewed and to have their survey data linked to their HDSS record.

## Results

### Characteristics of study population

Between February and June 2011, we enrolled 216 women who self-reported they were HIV-positive (173 from the HIV-positive HBCT sample and 43 from the random sample) and 318 women from the non-HBCT areas who self-reported being HIV-negative.

Among the 216 HIV-infected women, most (89.8%) were married and 51.9% had received at least primary level education (Table 1). Economic status was assessed by monthly income (40.2% of 82 women who responded reported a monthly income of at least US $25), ownership of a radio (64.8%), bicycle (59.7%) or cow (41.2%). The median number of children was 4 (IQR 3–6) with 54% of mothers reporting less than 5 prior births and 13.9% a prior pregnancy loss. Most (59.7%) HIV-infected women knew the HIV status of their partners, of whom 71.3% were HIV-infected. Age of study participants, employment status, mobile phone ownership, parity, ANC attendance, gestation at first ANC visit, number of ANC visits have been presented previously [20].

**Table 1:** **Baseline characteristics of study population (n = 534)**

**Characteristic**	**HIV-infected (n = 216)**		**HIV-uninfected (n = 318)**	
	**N(%) or median (IQR)**		**N(%) or median (IQR)**	
Age* (n = ^@^215)	29	(25–32)	25	(22–30)
Married*	194	(89.8)	299	(94.0)
> primary education* (n = ^317)	112	(51.9)	152	(48.0)
Employed (n = ^@^214; ^317)	77	(35.7)	141	(44.5)
Economic				
Income ≥ US $25 (n = ^@^82; ^150)	33	(40.2)	59	(39.3)
Don’t know	129	(59.7)	166	(52.2)
Missing	5	(2.3)	2	(0.6)
Own mobile phone* (n = ^317)	152	(70.4)	234	(73.8)
Own radio	140	(64.8)	236	(74.2)
Own bicycle	129	(59.7)	195	(61.3)
Obstetric				
Attend ANC*	209	(96.8)	301	(94.7)
Gestation at 1st ANC visit (months)* (n = ^@^215; ^294)	5	(4–6)	5	(4–6)
No of ANC visits* (n = ^@^202; ^282)	3	(3–4)	3	(3–4)
No of children	4	(3–6)	4	(3–6)
Para < 5	117	(54.2)	197	(62.0)
Pregnancy loss (n = ^317)	30	(13.9)	33	(10.4)
Partner				
Know partner status (n = ^178)	129	(59.7)	178	(56.0)
Partner HIV-infected	92	(71.3)	10	(5.6)
Very satisfied with delivery care (n = ^@^204; ^315)	135	(66.2)	210	(66.7)
Facility delivery	77	(77.0)	94	(74.6)

n = 216 among HIV infected and 318 among HIV-uninfected women unless otherwise indicated by ^@^ (HIV-infected) or ^ (HIV-uninfected).

*Previously reported [20].

Thirty-four percent of HIV-infected women reported at least one HIV-associated stigma indicator for shame, 86% reported at least one indicator for blame or judgment for people living with HIV/AIDS and 86% reported knowledge of at least one act of enacted stigma or discrimination in the previous one year due to HIV/AIDS [20].

Among the 318 HIV-uninfected women, most (94%) were married, 48% had received at least primary level education, 74.2% owned a radio and 50.4% a bicycle (Table 1). The median number of children was 4 (IQR 3–6) with 62% of mothers reporting less than 5 prior births and 10.4% a prior pregnancy loss. Most (56%) HIV-uninfected women knew the HIV status of their partners, of whom 5.6% were HIV-infected. Age of study participants, employment status, mobile phone ownership, parity, ANC attendance, gestation at first ANC visit, number of ANC visits have been presented previously [20]. Overall, 101 (46.8%) HIV-infected and 127 (39.9%) HIV-uninfected women delivered at health facilities.

### Correlates of facility delivery among HIV-infected women

Of 216 HIV-infected women enrolled, 101 (46.8%) delivered at health facilities. HIV-infected women who delivered at health facilities were more likely to have completed primary education (59.4% vs. 45.2%, p = 0.037) and be of higher socioeconomic status compared to those with non-facility delivery (57.5% vs. 23.8% had family monthly income of at least US $25, p = 0.002) and (80.2% vs. 61.7% owned a cell phone, p = 0.003) (Table 2). Although HIV-infected women who delivered at heath facility were more likely to own a radio (49.3% vs. 42.1%, p = 0.312), a bicycle (50.4% vs. 41.4%, p = 0.193), or a cow (50.6% vs. 44.1%, p = 0.348) compared to women with non- facility delivery, these rates were not statistically different between groups.

**Table 2:** **Comparison of selected characteristics between women who did not deliver at health facilities and those who did**

**Characteristic**	**HIV-infected (n = 216)**		**P value**	**HIV-uninfected (n = 318)**		**P value**
	**Non-facility (n = 115)**	**Facility (n = 101)**		**Non-facility (n = 191)**	**Facility n = 127)**	
	**N (%) or mean (95% CI)**			**N (%) or mean (95% CI)**		
Social demographics						
Age (years) (n = ^@^215)	29.1 (28.1–30.1)	28.6 (27.5–29.6)	0.444	26.6 (25.7–27.5)	25.6 (24.6–26.7)	0.158
Duration of resident in area(n = ^@^214; ^=316)	10.5 (9.3- 11.6)	9.6 (8.4–10.8)	0.294	9.0 (8.2- 9.9)	7.9 (6.8–9.0)	0.107
Married (n = ^@^214)	101 (87.8)	93 (92.1)	0.302	182 (95.3)	117 (92.1)	0.244
Employed (n = ^317)	39 (33.9)	38 (38.4)	0.497	77 (40.3)	64 (50.8))	0.066
≥ Primary education (n = ^317)	52 (45.2)	60 (59.4)	0.037	81 (42.4)	71 (56.4)	0.015
Economic						
Income ≥ US $ 25 (n = ^@^82; ^150)	10 (23.8)	23 (57.5)	0.002	28 (32.6)	31 (48.4)	0.049
Own mobile phone (n = ^317)	71 (61.7)	81 (80.2)	0.003	125 (65.5)	109 (86.5)	<0.005
Own radio	71 (61.7)	69 (68.3)	0.312	131 (68.6)	105 (82.7)	0.005
Own bicycle	64 (55.7)	65 (64.4)	0.193	110 (57.6)	85 (66.9)	0.094
Obstetric						
Number of pregnancies	4.7 (4.4–5.1)	4.3 (3.9–4.7)	0.079	4.6 (4.3–4.9)	3.8 (3.4–4.2)	0.002
Parity < 5	55 (47.8)	62 (61.4)	0.046	109 (57.1)	88 (69.3)	0.028
Prior pregnancy loss (n = ^317)	18 (15.7)	12 (11.9)	0.424	21 (11.1)	12 (9.5)	0.647
ANC attendance	109 (94.8)	100 (99.0)	0.080	175 (91.6)	126 (99.2)	0.003
Gestation at 1st ANC visit^#^ (n = ^@^205)	5.4 (5.2–5.7)	4.9 (4.6–5.2)	0.016	5.3 (5.1–5.6)	5.1 (4.9–5.4)	0.246
Very satisfied with care (n = ^@^204; ^315)	58 (55.8)	77 (77.0)	0.001	116 (61.4)	94 (74.6)	0.031
Know HIV positive status						
Before pregnancy	44 (38.3)	46 (45.5)	0.279	-	-	
During pregnancy	71 (61.7)	55 (54.5)		-	-	
Known partner HIV status	60 (52.2)	69 (68.3)	0.016	99 (51.8)	79 (62.2)	0.068
Partner HIV-infected (n = ^@^129; ^178)	43 (71.7)	49 (71.0)	0.935	6 (6.1)	4 (5.1)	0.774
Use of maternal ARVs (n = ^@^207)	89 (77.4)	88 (87.1)	0.063	-	-	
Use of infant ARVs (n = ^@^178)	83 (94.3)	88 (97.8)	0.275	-	-	
HIV stigma measures*						
Shame	41 (35.7)	33 (32.7)	0.645	82 (42.9)	60 (47.2)	0.449
Blame or judgment	102 (88.7)	83 (82.2)	0.173	173 (90.6)	111 (87.4)	0.370
Enacted stigma or discrimination	35 (87.5)	25 (83.3)	0.622	37 (84.1)	20 (76.9)	0.456

N = 216 among HIV infected (^@^ if different) and 318 among HIV-uninfected women (^ if different).

# Gestation in months.

*Woman agrees with at least one HIV related shame, blame or judgment, or enacted stigma or discrimination indicator.

HIV-infected women reporting facility deliveries were more likely to have initiated antenatal care earlier (mean 4.9 vs. 5.4 months, p = 0.016), be of lower parity (53.0% vs. 47.0% were less than para 5, p = 0.046), more likely to report having been very satisfied with care provided during delivery (77% vs. 55.8%, p = 0.001) and know the HIV status of their partner (68.3% vs. 52.2%, p = 0.016) than those with non-facility deliveries. There was a trend for women with facility deliveries to be more likely to use maternal antiretroviral drugs (87.1% vs. 77.4%, p = 0.063) compared to women with non-facility delivery. The proportion of women with history of prior pregnancy loss, who attended antenatal clinic, and used infant antiretroviral drugs did not differ between mothers who had facility versus non- facility deliveries. Measures of value and morality-related stigma and enacted stigma or discrimination did not differ by place of delivery.

In multivariate analysis, earlier initiation of antenatal care (OR = 1.24, 95% CI: 1.01–1.52) and knowledge of partner HIV status (OR = 1.89, 95% CI: 1.04–3.44) remained independently associated with facility delivery among HIV-infected women.

### Perceptions for reasons of non-facility delivery among HIV-infected women

Among HIV-infected women, cost of services was the most common (42.8%) reason given for women not giving birth at health facilities followed by perceived distance to facility (18.8%) and fear of harsh treatment by health providers (15.2%). Other reasons given were fear of HIV testing at health facility (2.9%), rapid labor so that delivery occurred before reaching the facility (2.9%), lack of transport (2.2%), perceptions that facilities were closed at night (1.4%) and fear of caesarean section (1.1%). Preference for traditional birth attendant was cited as reason for non-facility delivery in only 0.7% of responses.

### Correlates of facility delivery among HIV-uninfected women

To understand whether HIV-infected women had any determinants of facility delivery distinct from HIV-uninfected women, we assessed cofactors for facility delivery among 318 women from the random sample who reported they were HIV-uninfected. Of these, 127 (39.9%) delivered in a health facility. Similar to findings among HIV-infected women, HIV-uninfected women who delivered at health facilities were more likely to have completed primary education (56.4% vs. 42.4%, p = 0.015), and be of higher socioeconomic status compared to those with non-facility delivery (48.4% vs. 32.6% had family monthly income of at least US$25, p = 0.049; 86.5% vs. 65.5% owned a cell phone, p < 0.005 (Table 2). Antenatal clinic attendance (99.2% vs. 91.6%, p = 0.003) and number of children (mean 4.6 vs. 3.8, p = 0.002) were associated with facility delivery. Gestation at initiation of ANC, maternal age, employment, marital status, knowledge partner HIV status did not differ between HIV-uninfected women who delivered at facility and those who did not. Similar to findings among HIV infected, HIV-uninfected women who delivered at health facility were more likely to report being very satisfied with care provided during delivery (74.6% vs. 61.4%, p = 0.031) when compared to women with non-facility delivery.

In multivariate analysis, ownership of mobile phone (OR = 2.99, 95% CI: 1.6–5.57) and fewer number of children (OR = 0.84, 95% CI: 0.75–0.95 for each additional child) remained independently associated with facility delivery among HIV-uninfected women

### Perceptions for reasons of non-facility delivery among HIV-uninfected women

Similar to HIV-infected women, cost of services was the most common (38.6%) reason given by HIV-uninfected women for not giving birth at health facilities followed by perceived distance to facility (16.8%). Other reasons given were rapid labor so that delivery occurred before reaching the facility (10.5%), fear of harsh treatment by health providers (10.5%), fear of HIV testing at health facility (3.2%), perceptions that facilities were closed at night (2.7%) and fear of caesarean section (1.8%). Lack of awareness of benefits of facility delivery was cited as a reason in 3.2% of responses.

## Discussion

In this community-based study, we found that only 46.8% of HIV-infected and 39.9% of HIV-uninfected women delivered in a health facility. HIV-infected women stated that cost, distance, and fear of harsh treatment were primary disincentives for facility delivery, with <3% stating that fear of HIV testing was a disincentive. Among HIV-infected women, knowledge of partner HIV status, higher sociodemographic and educational status, and earlier ANC attendance were associated with facility delivery, however, markers of stigma were not. We identified specific groups of women at risk for non-facility delivery, and this may inform interventions to promote facility delivery. In contrast to some previous studies, we did not find that proportions of HIV-infected women who delivered at health facilities were lower than those of HIV-uninfected women [7,22]. Cofactors of facility delivery were similar between HIV infected and uninfected women suggesting that HIV-infected women may not have specific concerns that prevent them from accessing facility delivery.

The proportion of HIV-infected women who delivered at facility, though slightly higher than among HIV-uninfected women assessed in the same survey, was low and similar to that reported for women in general population in Nyanza Province, the region where the study was conducted [16]. Rates of facility delivery in Kenya have remained unchanged between 1993 and 2008 [16,23]. Facility delivery rate is a key indicator of progress towards attainment of MDG 5 because it is inversely correlated with maternal mortality ratio; and a 90% facility delivery rate is the 2015 MDG target [15]. Kenya missed the 2005 target (80% facility delivery) and is unlikely to attain the 2015 target. There is thus need for fresh strategies to increase facility delivery rates among both HIV-infected and HIV-uninfected women.

Among HIV-infected women, facility delivery offers an important opportunity to support PMTCT. The 2011 UNAIDS Global Plan for eliminating new HIV infections among children requires that 90% of HIV-infected women use perinatal antiretroviral drugs [4]. Facility delivery plays a critical role towards these efforts by facilitating use of antiretrovirals during labor. Consistent with other studies, we found that women who did not deliver at a health facility were less likely to use maternal antiretrovirals [5-7]. Facility delivery also provides an opportunity to employ obstetrical interventions to minimize duration of rupture of membranes and to prevent prolonged labor, both identified as risk factors for intrapartum HIV transmission [8,9]. For women who do not deliver at facilities, dispensing intrapartum antiretroviral doses antenatally or using PMTCT Option B regimens, where pregnant and breastfeeding women are offered antiretroviral therapy irrespective of CD4 counts, may decrease MTCT.

HIV infected-women who knew the HIV status of their partner were more likely to deliver in health facilities. This suggests a potential role for couple HIV counseling and testing to increase uptake of maternal child health services. Our findings are consistent with studies showing improved uptake of PMTCT interventions with male partner involvement [24,25]. Knowledge of partner status may reflect better communication among these couples enabling discussions on benefits of facility delivery. A recent study in Tanzania reported that facility delivery increased 2-fold when a couple discussed place of delivery during pregnancy [26]. There was a trend for association of knowledge of partner status with facility delivery in HIV-uninfected women, suggesting that knowledge of partner status may be a general marker of the strength of the couple’s relationship and potentially the male partner’s investment in the new infant. As the head of the family in most Kenyan homes, the male partner plays a key role in decision-making about the place of delivery and in provision of resources required to pay for costs of transport and maternity services.

In our study, women of lower economic status were less likely to deliver at health facilities, perhaps due to insufficient finances for transport or facility care. Higher rates of facility delivery have been reported with abolition of user fees for maternity services in several African countries [27-31]. In June 2013, the Kenya government abolished maternity user fees in all public health facilities. Free maternity policy will require efficient implementation to ensure capacity to cope with increased demand for services and provision of high quality services [28,32,33]. In addition, it is crucial to mitigate hidden costs such as unofficial provider or supply charges and travel costs that may discourage utilization of the free maternity services [34-36]. Other strategies to tackle financial barriers to facility delivery include demand-side health financing mechanisms such as vouchers or conditional cash transfer programs that cover user fees and related access costs, or supply side financing such as performance-based contracting and delivery incentive schemes [37-40]. In Cambodia, introduction of voucher and health equity funding schemes resulted in an almost 3-fold increase in facility deliveries [38]. A challenge of these financing programs is to ensure that poor and uneducated women, who have the greatest need, benefit [40]. In addition, they may be difficult to sustain.

Consistent with other studies, we found that higher education levels among women were associated with facility delivery irrespective of HIV status [41-44]. In the 2008 KDHS survey, among mothers with no education, only 15% had facility delivery in contrast to 72% facility delivery rate among mothers with a secondary or higher education [16]. Women with higher levels of education are likely to be aware of dangers of non–facility delivery and may be more receptive to health information.

Most women in our study made at least one ANC visit providing opportunity to interact with health workers and learn the importance of facility delivery. We found that HIV-infected women who initiated antenatal care earlier were more likely to deliver at health facilities, consistent with some previous studies [26,45]. Early ANC attendance may reflect existing trust in the health care system. Alternatively, increased contact with health workers may provide opportunity to educate women on importance of facility delivery and reinforce these messages. Mobile phone technology can be used as appointment reminders or to reinforce health messages to improve maternal outcomes and may exert a similar effect on facility delivery if health education is responsible for the observed associations between ANC visits and delivery location [46]. The high prevalence of mobile phone ownership may thus provide an opportunity to employ *mHealth* technology to promote earlier antenatal care and facility delivery.

Intriguingly, rates of facility delivery among HIV-infected did not differ by stigma measures, marital status, prior pregnancy loss or maternal age. Fear of HIV-related stigma and discrimination has been reported to discourage HIV-infected mothers from delivering in health facilities [47]. The proportion of HIV-infected mothers reporting at least one HIV related blame, judgment or enacted stigma indicator in our study did not differ between HIV–infected mothers who delivered at health facility and those who did not. However, these measures of stigma are not comprehensive and it is possible that qualitative studies would reveal aspects of stigma contributing to non-facility delivery that we could not assess in this survey.

Our study has several strengths. The population-based approach allowed us a unique opportunity to access mothers who did not utilize MCH services; these mothers would have been missed if the study had been facility-based. Working within the HDSS area where home-based HIV counseling and testing had been conducted enabled oversampling of HIV-infected women, providing an opportunity to explore HIV as a correlate of facility delivery with a relatively small sample size. Limitations of the study included dependency on self-report for place of delivery and the cross-sectional design. Most women did not know monthly family income and surrogate measures such as ownership of phones or radios that vary in costs may be a suboptimal proxy for economic status.

## Conclusion

The low utilization of skilled care at birth poses a serious challenge to attainment of the 5th Millennium Development Goal and of the 2015 Global Plan goal to eliminate pediatric HIV. Our findings suggest that promoting earlier antenatal care and engaging partners may contribute both to decreased infant HIV infection and better general maternal and child outcomes.
